# Supplementary figures and images for: Caveolae couple mechanical stress to integrin recycling and activation
Source: eLife. 2022 Oct 20;11:e82348. doi: 10.7554/eLife.82348 (PMC9747151; doi:10.7554/eLife.82348)

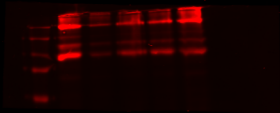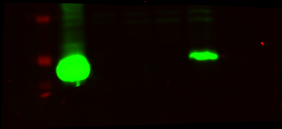

Supplement: Figure 1—source data 1. [file elife-82348-fig1-data1.pdf]

Cav1KO

Cav1WT

Cav1KO

PTRF

Empty

Cav1

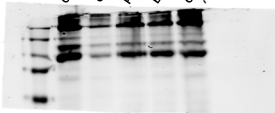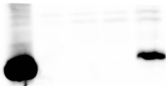

Supplement: Figure 1—source data 2. [file elife-82348-fig1-data2.pdf]

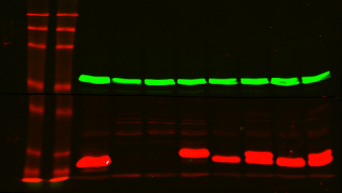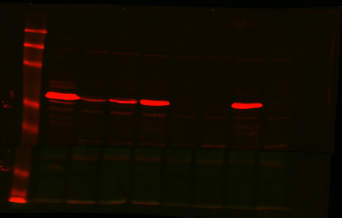

Supplement: Figure 1—source data 3. [file elife-82348-fig1-data3.pdf]

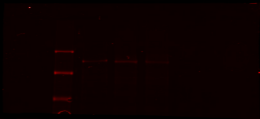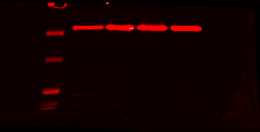

Supplement: Figure 3—figure supplement 1—source data 1. [file elife-82348-fig3-figsupp1-data1.pdf]

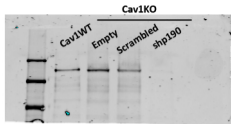

**Mouse anti p190RhoGAP**  
**Upstate 1:1000**

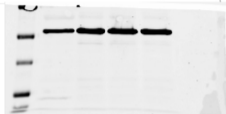

**Mouse anti-alpha tubulin**  
**Abcam 1:10.000**

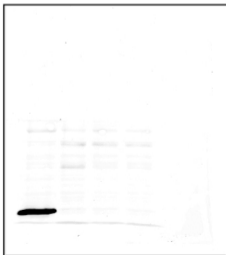

**Rabbit anti cav1**  
**(CS) 1:1000**

Supplement: Figure 3—figure supplement 1—source data 2. [file elife-82348-fig3-figsupp1-data2.pdf]

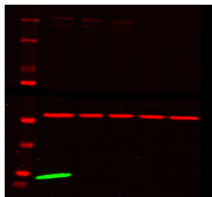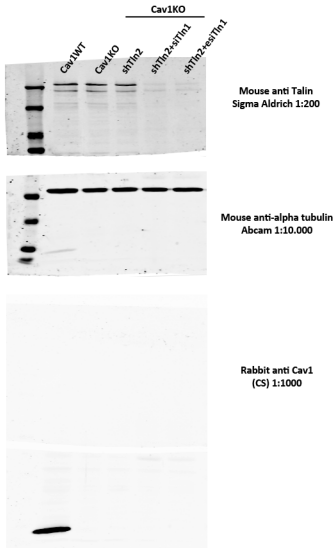

Supplement: Figure 7—figure supplement 2—source data 2. [file elife-82348-fig7-figsupp2-data2.pdf]

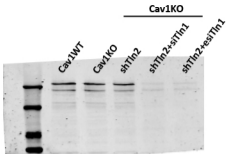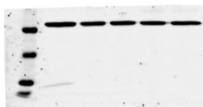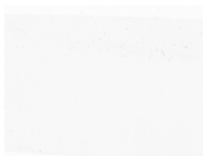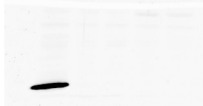

Supplement: Figure 7—figure supplement 2—source data 3. [file elife-82348-fig7-figsupp2-data3.pdf]
