# Supplementary material for: Caveolae couple mechanical stress to integrin recycling and activation
Source: eLife. 2022 Oct 20;11:e82348. doi: 10.7554/eLife.82348 (PMC9747151; doi:10.7554/eLife.82348)
Supplement: Figure 1—source data 4. [file elife-82348-fig1-data4.pdf]

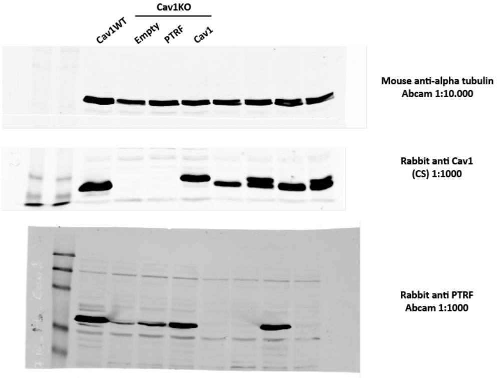

Cav1KO
Cav1WT
Empty
PTRF
Cav1
Mouse anti-alpha tubulin
Abcam 1:10.000
Rabbit anti Cav1
(CS) 1:1000
Rabbit anti PTRF
Abcam 1:1000
